# 

**Published:** 1894-02

**Authors:** 


### CONTENTS.

ORIGINAL COMMUNICATIONS—
Foreign Bodies in Pharynx and Larynx. By Arthur G. Hobbs and George Brown, M.D., Atlanta, Ga. .... 705
External Urethrotomy Versus Internal. By W. C. Dugan, M.D., Louisville, Ky. 709
A Case of Chronic Cystitis and Urethritis with Ulcers. By C. D. Hurt, M.D., Atlanta, Ga. .... 715
A Case of Syphilis by Conception. By Prof. Chambrelent, Bordeaux, France.... 717
Injuries to the Spine. By J. Y. H. Smith, M.D., Rochelle, Ga. .... 722
SOCIETY REPORTS—
Atlanta Society of Medicine .... 724
Macon Medical Society .... 729
EDITORIAL—
The Medical Buccaneer .... 736
From a Sister State .... 737
SELECTIONS AND ABSTRACTS—
Skin Diseases and Syphilis .... 739
Gynecology and Obstetrics .... 748
Surgery .... 751
Practical Chemistry and Pharmacy .... 761
MEDICAL ITEMS .... 764
BOOK REVIEWS .... 767
ADVERTISERS' INDEX TO ADVERTISEMENTS .... xii
CLASSIFIED INDEX TO ADVERTISEMENTS .... xxxix
ITEMS OF INTEREST .... x, xi, xxxvii, xxxviii
JOURNAL FOR 1894 .... xxii
PREMIUM OFFERS .... xxxvi

